# Redox Regulation in Aging Lungs and Therapeutic Implications of Antioxidants in COPD

**DOI:** 10.3390/antiox10091429

**Published:** 2021-09-07

**Authors:** Hirofumi Kiyokawa, Yuma Hoshino, Kazuhiro Sakaguchi, Shigeo Muro, Junji Yodoi

**Affiliations:** 1 Center for Regenerative Medicine, Boston Medical Center, Boston University, Boston, MA 02118, USA; hirofumi@bu.edu; 2 Kakeyu-Misayama Rehabilitation Center, 1308 Kakeyu-onsen, Ueda, Nagano 386-0396, Japan; yumahoshino@gmail.com; 3 Department of Respiratory Medicine, Nara Medical University, 840 Shijo-cho, Kashihara, Nara 634-8522, Japan; k-sakaguchi@naramed-u.ac.jp; 4 Japan Biostress Research Promotion Alliance (JBPA), 1-6 Shogoin Kawahara-cho, Sakyo-ku, Kyoto 606-8397, Japan; yodoi@skyblue.ocn.ne.jp; 5 Institute for Virus Research, Kyoto University, 53 Shogoin Kawahara-cho, Sakyo-ku, Kyoto 606-8507, Japan

**Keywords:** COPD, thioredoxin, antioxidant, oxidative stress, aging

## Abstract

Mammals, including humans, are aerobic organisms with a mature respiratory system to intake oxygen as a vital source of cellular energy. Despite the essentiality of reactive oxygen species (ROS) as byproducts of aerobic metabolism for cellular homeostasis, excessive ROS contribute to the development of a wide spectrum of pathological conditions, including chronic lung diseases such as COPD. In particular, epithelial cells in the respiratory system are directly exposed to and challenged by exogenous ROS, including ozone and cigarette smoke, which results in detrimental oxidative stress in the lungs. In addition, the dysfunction of redox regulation due to cellular aging accelerates COPD pathogenesis, such as inflammation, protease anti-protease imbalance and cellular apoptosis. Therefore, various drugs targeting oxidative stress-associated pathways, such as thioredoxin and N-acetylcysteine, have been developed for COPD treatment to precisely regulate the redox system. In this review, we present the current understanding of the roles of redox regulation in the respiratory system and COPD pathogenesis. We address the insufficiency of current COPD treatment as antioxidants and discuss future directions in COPD therapeutics targeting oxidative stress while avoiding side effects such as tumorigenesis.

## 1. Introduction

Mammals, including humans, are aerobic organisms that have a mature respiratory system for the uptake of oxygen (O_2_) and release of carbon dioxide (CO_2_) that maintains life. Whereas oxygen is vital for the generation of adenosine triphosphate (ATP), the universal energy donor in the mitochondria of every cell, excessive reactive oxygen species (ROS), the byproducts of aerobic metabolism, contribute to the development of a wide spectrum of pathological conditions in various organs from the reproductive system [1] to the nervous systems [2]. In addition to endogenous ROS, epithelial cells in respiratory systems can be directly exposed to and challenged by exogenous ROS, including ozone (O_3_) and cigarette smoke, which enhances oxidative stress by generating secondary ROS, such as superoxide radicals (O_2_^−^) and hydrogen peroxide (H_2_O_2_), on the respiratory surface [3]. For tissue homeostasis, especially in the respiratory system, antioxidant defense mechanisms play essential roles in the tight regulation of ROS levels.

**Figure 1:**
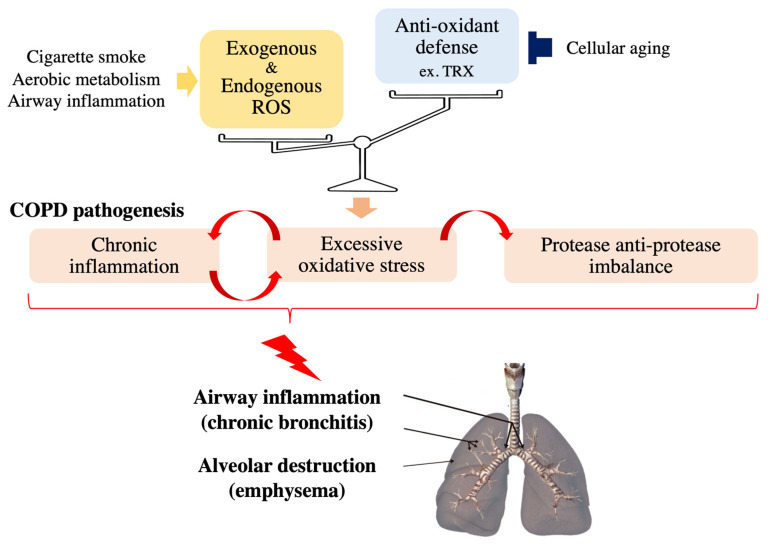
Effects of oxidative stress on COPD pathology. Noxious oxidative stress occurs and contributes to the progression of COPD pathogenesis, such as chronic inflammation and protease anti-protease imbalance, when cellular reactive oxygen species (ROS) levels overwhelm antioxidant capacity.

Chronic obstructive pulmonary disease (COPD) is currently the third leading cause of death worldwide and is characterized and defined by the progression of small airway remodeling (chronic bronchitis) and alveolar destruction (emphysema), which causes expiratory airflow limitation and results in chronic respiratory symptoms, including dyspnea, persistent cough and phlegm (Figure 1) [4]. A leading cause of COPD is chronic exposure to cigarette smoke and biomass fuel, since a single puff of cigarette smoke contains as many as 4 × 10^14^ free radicals [5]. These ROS damage small airways and lung parenchyma directly or indirectly by enhancing inflammation and excessive proteases in the lungs of patients with COPD [4,6]. In addition, lung aging increases the risk and accelerates the progression of COPD [7] because it impairs various basic cellular processes responsible for COPD development including the antioxidant defense mechanisms [8,9]. Noxious oxidative stress in combination with exacerbated systemic inflammation also contributes to the extrapulmonary comorbidities, including osteoporosis, which negatively affects the quality of life and prognosis of patients with COPD. Since currently available COPD treatment has a very limited beneficial effects on oxidative stress, several drugs targeting oxidative stress-associated pathways, such as thioredoxin (TRX) and N-acetylcysteine (NAC), have been developed for COPD treatment to regulate the redox system precisely [6,10,11,12] to ameliorate the molecular pathogenesis of COPD.

In this review, we present the current understanding of the roles of redox regulation in the respiratory system and COPD pathogenesis, in which impaired oxidative balance due to impairment and/or overwhelming by ROS plays a central role. Since previous studies have revealed that lung aging contributes to COPD susceptibility and progression, the interactive association between aging and impaired redox balance are also addressed. Finally, we discuss future treatment strategies with a focus on how to correct excessive ROS levels, which remains still undertreated because of the limited efficacy of conventional COPD treatment to control oxidative stress. We will focus on TRX because it has antioxidant and anti-inflammatory effects and can be used as a noninvasive maker to guide antioxidant treatment to avoid the long-term side effects such as tumorigenesis.

## 2. Redox Regulation in the Mammalian Respiratory System

### 2.1. The Sources and Roles of Oxidative Stress in the Respiratory System

Despite the direct and continuous exposure to high concentrations of oxygen and environmental pollutants such as O_3_, NO_2_ and cigarette smoke, the functions and structures of the respiratory system are robustly maintained via the tight regulation of ROS levels by antioxidant systems [3,6,11]. In addition to exogenous ROS, ROS are endogenously generated by biological processes such as aerobic metabolism in the mitochondria [13,14]. Enzymatic reactions in the mitochondrial electron transport chain generate superoxide radicals (O_2_^−^), which are dismutated to a more stable and uncharged form, hydrogen peroxide (H_2_O_2_), by superoxide dismutase (SOD) [15]. As a result, H_2_O_2_ can leave the mitochondrion to mediate various cytosolic cell signaling pathways. Accumulating evidence has demonstrated that H_2_O_2_ in combination with ROS produced by other endogenous sources, such as peroxisomes and cyclooxygenases, plays essential roles in several vital cellular processes, such as cellular proliferation, senescence, apoptosis, and ECM remodeling [16,17,18]. ROS affect the regulation of numerous signaling pathways through the oxidation of amino acid residues in the protein chain, which changes the protein structure and function. Cysteine (Cys) is one of most sensitive residues to ROS, and its oxidative reaction is mainly mediated by two major redox machineries, glutathione (GSH) peroxidase (GPX) and thioredoxin (TRX) (see detailed information about GPX in Section 2.2 “Antioxidant defense system” and TRX in Section 4.1 “Thioredoxin (TRX)”) [11,19,20,21,22]. Thus, ROS work as “second messengers” in physiological intracellular signaling pathways.

In contrast to the essential roles of ROS in normal biological functions, excessive ROS levels result in multiple diseases, such as cancers and COPD, by impairing cellular functions and inducing cellular aging and death [6,23,24]. This dose-dependent behavior of ROS is described as “hormesis”, an important concept for better understanding the roles of ROS in vivo; low levels of oxidative stress are essential for cellular homeostasis, such as the initiation of cytoprotective programs, whereas high oxidative stress is toxic [9,25,26].

Redox regulation in the respiratory system can be overwhelmed by the reciprocal interactions between inflammation and oxidative stress (Figure 1). Continuous exposure of respiratory epithelial cells to pathogens such as bacteria and viruses induces the recruitment of inflammatory cells (mainly macrophages and neutrophils), which results in the enhancement of ROS levels in the respiratory system; activated neutrophils and macrophages are important endogenous sources of respiratory system ROS [23] Activated neutrophils secrete myeloperoxidase (MPO), which is the only enzyme in the body that produces the extremely destructive hypochlorous acid (HOCl) by chlorinating the tyrosine residues of proteins [24]. The levels of chlorinated tyrosine (namely, 3-chlorotyrosine) are significantly increased in the sputum of patients with COPD, demonstrating that activated neutrophils are key effector cells in COPD [25]. Increased oxidative stress, in turn, strengthens inflammation by activating NF-κB and p38 MAPK, which induce the expression of inflammatory genes such as IL-8 and TNF-α, important cytokines in COPD, leading to airway inflammation and emphysematous changes in patients’ lungs [25,26,27]. In addition, epithelial cells exposed to oxidative stress also release proinflammatory cytokines [28]. Supporting the notion that p38 MAPK is one of the key mediators in the pathogenic interaction between inflammation and cigarette smoke (CS)-induced oxidative stress, Marumo S et al. demonstrated that the activation level of p38 determines susceptibility to CS-induced emphysema [29]. This study also showed that intraperitoneal injection of SB203580, a p38 MAPK inhibitor, into CS-exposed mice ameliorates lung inflammation and injury. However, under normal conditions, tissue homeostasis can be robustly maintained due to the antioxidant defense mechanisms in healthy cells, which are capable of efficiently removing these detrimental ROS to avoid pathological conditions.

### 2.2. The Antioxidant Defense System

**Figure 2:**
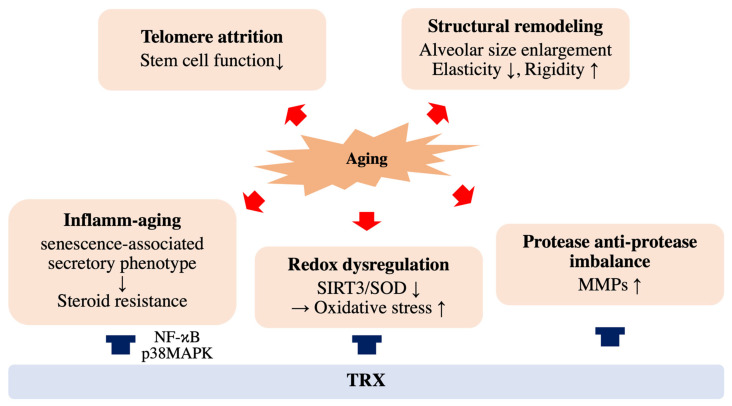
Effects of aging and thioredoxin (TRX) on COPD pathology. Cellular aging accelerates COPD progression through structural remodeling, telomere attrition, inflammatory aging, redox dysregulation and protease and anti-protease imbalance. TRX has therapeutic potential for COPD by improving phenomena such as inflamm-aging and redox dysregulation.

Damaging oxidative stress occurs when cellular ROS levels overwhelm antioxidant capacity. To tightly regulate oxidative stress levels, the respiratory system, including airway epithelial cells, possesses antioxidant machinery that protects against high oxygen tension, and endogenous and exogenous ROS. There are two types of antioxidant defense systems, enzymatic and nonenzymatic. Enzymatic antioxidants include superoxide dismutase (SOD), catalase, glutathione peroxidase (GPX), peroxiredoxins (PRX) and thioredoxin (TRX) [3,6,30]. First, SODs such as SOD3, which is highly expressed in type 2 alveolar and bronchial epithelial cells in the lungs, catalyze the conversion of superoxide radicals to H_2_O_2_ [23]. H_2_O_2_ is mainly broken down by catalase and GPX into H_2_O and O_2_. Although catalase is expressed in most of the cells in aerobic animals, it is most prominently localized in type 2 alveolar epithelial cells and macrophages in the respiratory system [23]. GPXs are a family of selenium-dependent and selenium-independent antioxidant enzymes that reduce H_2_O_2_ to H_2_O by oxidizing GSH [23]. GPX1 is the predominant GPX and is expressed in airway/alveolar epithelial cells and alveolar macrophages [31]. PRX also breaks down H_2_O_2_ and hydroperoxides and all six members of the mammalian PRX family are expressed in different areas of the lungs, protecting against exogenous and endogenous ROS [32,33]. Thioredoxin (TRX) is a redox-acting small protein that is well conserved across species from plants to mammals [30]. In addition to the ability of TRX itself to scavenge singlet oxygen or hydroxyl radicals [34], it also works as a radical scavenger in cooperation with PRX, as does the GSH system (Figure 2) (see the detailed information about TRX in Section 4.1 “Thioredoxin (TRX)”) [35]. Small nonenzymatic low-weight molecules, including uric acid [36] and dietary antioxidants, such as vitamin C (ascorbic acid) [37] and vitamin E (α-tocopherol) [38], also contribute to redox regulation in the lungs. Oxidative stress induces these antioxidant genes through the regulation of transcription factors such as nuclear factor E2-related factor 2 (Nrf2) and Forkhead box class O3a (FoxO3a). Nrf2 regulates the transcription of several antioxidant genes that encode many of the aforementioned enzymes. The function of Nrf2 is typically inhibited by ubiquitination via Kelch-like ECH-associated protein 1 (Keap-1). However, under oxidative stress, Nrf2 dissociates from Keap-1 and is transported to the nucleus, activating the transcription of antioxidant genes [39,40]. Oxidative stress also activates the transcription factor FoxO3a, which further induces the transcription of antioxidant genes [41]. Thus, these transcription factors constitute negative feedback loops to balance the redox level in response to ROS.

## 3. Impaired Redox Regulation in Aging and Patients with COPD

When antioxidant defense mechanisms are compromised and/or overwhelmed by cellular aging and enhanced oxidative stress due to the pathogenic reciprocal interaction between inflammation and ROS, chronic pulmonary diseases, including COPD, can occur (Figure 1). In this section, we provide an overview of how the redox dysregulation in the respiratory system that occurs with aging contributes to COPD pathogenesis, shedding light on possible future treatments for COPD.

### 3.1. Aging Is a Major Risk Factor for Chronic Lung Diseases, Including COPD

Physical and cellular aging is a major risk factor for chronic lung diseases, such as COPD, ILD and lung cancers [42,43]. Lung aging is associated with structural remodeling and declining respiratory functions [44] and results in the enlargement of alveolar size, similar to the emphysematous changes found in COPD [45]. Lungs gradually lose their elasticity with aging and become more rigid due to altered expression of extracellular matrix proteins, such as laminin, elastin and fibronectin. Thus, when cellular senescence is induced in mice, such as in SAM-P/1 [46] and Klotho knockout [47] mouse lines, characteristics of aging lungs arise, such as airspace enlargement. Klotho protein prevents the cellular damage and apoptosis by ameliorating oxidative damage and local inflammation [48,49]. In fact, Klotho expression is decreased in the lungs of smokers and further reduced in patients with COPD [49], and its plasma concentration in patients with COPD remained unchanged despite the functional improvement by the rehabilitation program [50]. These findings suggest that accelerated aging predisposes the lungs to COPD [7]. On the other hand, a recent study based on the long-term COPD cohort studies demonstrated that there are two distinct types of trajectories in terms of the respiratory functional declines; One subtype shows a rapid decline in FEV_1_ from a normal level of lung function in early adulthood, while the other shows a rather normal decline in FEV_1_ but from a low initial value of FEV_1_ [51]. The existence of the latter subpopulation underscores the importance of abnormal lung development or severe tissue injury in early adulthood in COPD pathogenesis. Therefore, it is noteworthy to know that accelerated lung aging is one of the major causes for COPD development.

Cellular aging is defined by classic hallmarks, including genomic instability, telomere attrition, epigenetic alterations, loss of proteostasis, deregulated nutrient sensing and mitochondrial dysfunction [52]. Oxidative stress is also thought to accelerate telomere attrition [53], which is negatively associated with an increased incidence of diseases and poor survival outcomes [54]. Previous studies demonstrated that telomere length is significantly shorter in patients with COPD than in healthy controls and is associated with the progression of severe emphysema after CS exposure [55,56]. Consistent with these findings, the induction of murine telomere dysfunction in type 2 alveolar epithelial cells, a stem cell population in lung alveoli, recapitulates the regenerative failure seen in patients with ILD or COPD [57].

Older adults exhibit elevated levels of proinflammatory cytokines, such as IL-1b, IL-6 and TNF-α, in both the serum and in lung tissues [58,59,60,61,62]. This enhanced inflammatory state is described as “inflamm-aging” or “inflammaging” [63], which results from a gradual increase in senescent cells of both immune and nonimmune origins (Figure 2). Recent studies have demonstrated that senescent cells secrete several secretory proteins, such as inflammatory cytokines, chemokines, growth factors and matrix metalloproteinases (MMPs), which damage the cells in the lung niche responsible for normal tissue repair [64,65]. This phenotypic characteristic of senescent cells, termed the senescence-associated secretory phenotype (SASP), contributes to the susceptibility of aging lungs to CS-associated injury. p38MAPK, which is relevant to COPD pathogenesis, plays an essential role in the development of SASP by activating NF-κB [66], which is also responsible for COPD progression by activating proinflammatory genes encoding cytokines and chemokines, such as IL-1b/IL-8/TNF-a, and enhancing oxidative stress [31,67,68]. Moreover, enhanced p38MAPK expression is associated with glucocorticoid resistance in COPD treatment through the phosphorylation of glucocorticoid receptor (GR) [69,70,71]. In addition, increased secretion of MMPs by senescent cells leads to an imbalance between proteases and antiproteases in the alveoli and directly damages alveolar structures, which is a central component of COPD development [67,72]. This imbalance between protease and antiprotease in COPD pathogenesis is supported by the fact that deficiency in alpha-1 antitrypsin (A1AT), a protein that prevents enzymes such as trypsin and MMPs from degrading normal tissue, is one of the highest risk factors for COPD [68,73].

### 3.2. Redox Dysregulation in Aging Lungs

Since mitochondrial dysfunction, one of the main sources of endogenous ROS, is one of the distinct hallmarks of cellular aging [52], the notion that progressive mitochondrial dysfunction with aging results in increased ROS production and accelerates cellular aging was proposed in the 1950s [74,75]. However, this notion, referred to as “the mitochondrial free radical theory of aging”, has been intensively re-evaluated since 2000 [76]. Unexpectedly, increased ROS have been demonstrated to prolong lifespan in yeast and *C. elegans* [77,78]. In addition, increased mitochondrial ROS does not lead to accelerated aging [79]. In contrast, SIRT3, which is an NAD^+^−dependent protein deacetylase that regulates the production of ROS in mitochondria, was demonstrated to prolong human lifespan by reducing ROS production through deacetylating SOD, a major mitochondrial antioxidant enzyme [80]. Consistent with this finding, Sirt3 and SOD protein expression is significantly decreased in the airway epithelia of CS-induced COPD rats and in the lungs of aged mice [81], while Sirt3 overexpression protects mitochondria from oxidative damage in CS extract-treated human airway epithelial cells by decreasing SOD acetylation [82]. These findings also reflect the dose-dependent behavior of ROS, “hormesis”, in the respiratory system. This notion has been shown by Paul MK et al. in mouse and human basal cells, a stem cell population of the large airways [83]. This group demonstrated that intracellular flux from low to moderate ROS levels is required for the induction of stem cell self-renewal and proliferation, while Nrf2 activated by increased ROS levels, an antioxidant that scavenges intracellular ROS, returns overall ROS levels to a low state to prohibit the excessive proliferation of basal cells. Since Nrf2 function is compromised during the aging process [84], the impaired function of respiratory stem cell populations due to the upregulation of ROS levels with aging predisposes the lungs to chronic pulmonary diseases such as COPD.

### 3.3. Redox Dysregulation in COPD Patient Lungs

Key transcription factors, such as Nrf2 and FoxO3, that activate antioxidant genes are decreased in COPD patient lungs [85,86,87]. Accumulating evidence indicates that increased oxidative stress within the local lung microenvironment is a major driving mechanism in COPD pathology [3,6,88]. The analysis of specimens such exhaled breath and its condensate, which can be obtained noninvasively, has revealed that oxidative stress, including ethane [89], H_2_O_2_ [90] and 8-isoprostanes [91], are significantly increased in patients with COPD compared to those in normal individuals. As discussed above, inflammatory signaling, such as that via NF-κB and p38MAPK, is controlled by ROS, and an increase in oxidative stress enhances local inflammation in COPD patient lungs through the recruitment of neutrophils and macrophages [92], resulting in the acceleration of COPD progression.

8-Isoprostanes are frequently investigated biomarkers of oxidative stress and markers of COPD severity [93]. The production of markers for oxidative stress, including 8-isoprostanes, is significantly enhanced during disease exacerbation [88,94]. Frequent acute exacerbations of COPD, defined as an acute worsening of respiratory symptoms that results in additional therapy [4], have been demonstrated to be associated with COPD progression and poor patient prognosis in cohort studies [95,96]. In accordance with these, GSH level, a marker of antioxidant status, is significantly lower in bronchoalveolar lavage fluid from the patients with COPD during acute exacerbations than under stable condition [97]. We directly proved that acute COPD exacerbations accelerate emphysematous changes in COPD patient lungs by the detailed analysis of lung parameters with time-series chest CT scans [98]. Acute exacerbations of COPD are mainly triggered by respiratory viral infection, while bacterial infections and environmental factors such as pollution may also initiate and/or amplify these events [4,99]. Enhanced inflammation triggered by viral infection during exacerbations plays central roles in COPD progression. We demonstrated that mimicking viral infection in CS-exposed mice by administering polyinosine-polycytidylic acid [poly(I:C)], an agonist of virus-induced innate immunity, accelerated emphysematous progression by enhancing airway neutrophilic inflammation, oxidative stress and lung apoptosis [100]. This study demonstrated the pathogenic interactions between inflammation and ROS during COPD exacerbations. Moreover, this study also underscores the importance of thioredoxin (TRX), a redox-acting small protein, in the treatment of COPD exacerbations because it ameliorates exacerbation-related emphysema progression by inhibiting acute and late neutrophil inflammation, which is resistant to the standard dose of dexamethasone used in clinical practice. Local ROS are also involved in the imbalance between proteases and antiproteases because oxidative stress inactivates antiproteinases such as alpha-1 antitrypsin (A1AT) and secretory leukocyte protease inhibitors while activating MMPs [101,102]. Thus, targeting redox regulation in COPD pathogenesis is a possible treatment option to inhibit excessive ROS, inflammation and proteases.

In addition to the negative effects of oxidative stress in the lungs, increased systemic ROS are detected, especially during COPD exacerbations, which are indicated by the increase in lipid peroxidation products and 4-hydroxynonenal (4-HNE) in the serum [103,104]. Although the detailed mechanisms underlying the comorbidities of COPD remain to be elucidated, ROS in combination with systemic inflammation are involved in the pathology of extrapulmonary manifestations, such as osteoporosis and skeletal muscle weakness, in patients with COPD [105]. Higher levels of oxidative stress are detected in the skeletal muscle of patients with COPD [106]. In accordance with this, we demonstrated that acute exacerbations were significantly associated with extrapulmonary manifestations such as osteoporosis and skeletal muscle weakness in a prospective cohort study [107,108]. These results suggest that the fine-tuning of redox regulation may prevent extrapulmonary comorbidities in patients with COPD. (Additionally, see the excellent comprehensive review for the mechanisms and targeted drug therapy for COPD written by Wang C et al. in 2020 [22]).

## 4. Antioxidant Drugs

Effective drug therapies to ameliorate respiratory symptoms and delay disease progression have been developed for patients with COPD and are mainly composed of long-acting bronchodilators with or without inhaled corticosteroids (ICSs) [4]. Acute exacerbations can be partially managed with systemic administration of corticosteroids in combination with antibiotics [4]. However, these treatment options are insufficient to stop disease progression and normalize impaired lung structures and functions. Corticosteroids are frequently administered in clinical medicine as a powerful and universal anti-inflammatory treatment; however, oxidative stress also contributes to steroid resistance, one of the key issues in COPD treatment. The function of histone deacetylase-2 (HDAC2), essential for the suppression of inflammatory genes, is impaired in patients with COPD due to oxidative stress [6,70]. To Y et al. demonstrated that corticosteroid insensitivity in patients with COPD is dependent on the activation of phosphoinositide-3-kinase-delta (PI3K-δ) due to oxidative stress [109]. PI3K-δ phosphorylates and ubiquitinates HDAC2, which prevents the corticosteroid from switching off activated inflammatory genes. The results of these studies suggest that precise control of the PI3K-HDAC2 axis can improve steroid resistance due to oxidative stress.

Since oxidative stress plays pivotal roles in several COPD pathogeneses, such as inflammation, protease imbalance and steroid resistance, as discussed above (Figure 1), redox-based drugs targeting detrimental oxidative stress in patients with COPD have been enthusiastically investigated in recent decades [6,11,22,110]. Many drugs, including TRX and N-acetylcysteine (NAC), have been tested in in vivo animal models and in clinical studies [6]. Animal studies, such as those in CS-exposed mice, including our study conducted in Kyoto university, have demonstrated that antioxidant treatment can significantly delay emphysema progression by decreasing inflammation and oxidative stress [100,111]. However, clinical studies have revealed only partial responses to antioxidant drugs, such as a decrease in exacerbation frequency in subpopulations, partly because of the heterogeneity of patients with COPD and the dose-dependent behavior of ROS in vivo. In this section, we will discuss the efficacy and limitations of redox-targeting treatment with a focus on the well-studied antioxidants TRX and NAC.

### 4.1. Thioredoxin (TRX)

**Figure 3:**
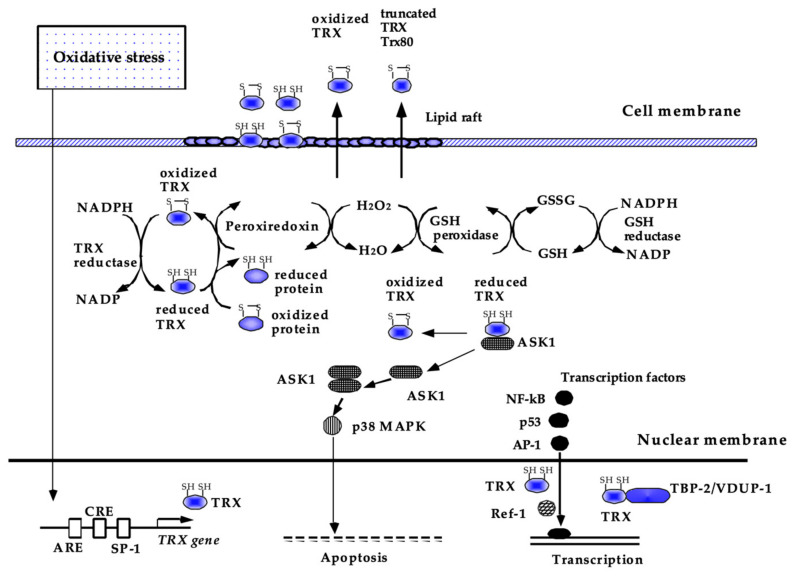
Current understanding of the biological functions of TRX. Thioredoxin (TRX) is a redox-acting protein that exchanges disulfide to dithiol to maintain the reducing status of various molecules. In the cytoplasm, TRX acts as a radical scavenger, either by itself or in cooperation with peroxiredoxin. TRX also has antiapoptotic and anti-inflammatory effects, some of which are attributed to the regulation of intracellular signal transduction, such as ASK-1 and p38 MAPK, and DNA binding of NF-kB, AP-1, and p53. TRX is ubiquitously present in the human body and is also inducible by a wide variety of stress conditions via the modulation of transcription factor binding to its promoter region. This figure has been reproduced with permission from the authors [21].

Human TRX is a 12-kDa small protein consisting of 105 amino acids with a conserved CXXC construct in its active site, which exchanges disulfide to dithiol to maintain the reducing status of various molecules. As shown in (Figure 3), the reducing activity of TRX is maintained by NADPH and thioredoxin reductase [112]. TRX is ubiquitously present in the human body and is also induced by a wide variety of stress conditions, including viral infection [113,114]. In addition to the ability of TRX to scavenge singlet oxygen or hydroxyl radicals [34], TRX has anti-inflammatory and anti-apoptotic properties via the regulation of intracellular signal transduction [115,116]. TRX inhibits ASK-1 and p38MAPK to suppress apoptosis [110,117] and regulates key transcription factors, such as NF-κB, AP-1, and p53 [118,119,120]. The activity of TRX is regulated by thioredoxin-binding protein-2 (TBP-2), also known as thioredoxin-interacting protein or vitamin-D3–upregulated protein-1 (VUDP-1) [121].

Since TRX is secreted from cells in response to oxidative stress and macrophage migration inhibitory factor (MIF), a classic inflammatory cytokine and another member of the TRX family [122], it has been assumed to have therapeutic potential for several diseases in which oxidative stress, inflammation and apoptosis are involved, such as COPD. Its effectiveness as a therapeutic protein has been reported in many animal models either by using TRX transgenic mice [123,124,125] or by injecting recombinant TRX [126,127,128]. Consistently, we have also demonstrated the beneficial effects of TRX on CS-induced emphysema using a mouse model [100,111]. TRX ameliorates progressive lung destruction in COPD by blocking the pathogenic interactions between oxidative stress and inflammation by inhibiting key inflammation-related transcription factors, such as p38 MAPK and NF-kB, and by directly scavenging oxygen free radicals (Figure 2). Moreover, TRX improves the imbalance between proteases and antiproteases by inhibiting the activities of tissue inhibitors of metalloproteinases (TIMPs) [129]. Consistent with this, TRX prevents the development and progression of elastase-induced emphysema in mice [10].

Taking into account these beneficial effects of TRX, such as anti-inflammatory effects, we planned to test the efficiency of TRX as a treatment option for acute lung injury and acute respiratory distress syndrome (ALI/ARDS) in a clinical cohort study at Kyoto University. However, the study has been suspended because of the shortage of drug substances. Since COVID-19, in which cytokine storm is a central pathology [130], is one of the largest current issues worldwide, TRX efficacy in COVID-19 treatment must be assessed in a large cohort study. Acute exacerbation is also a good indication for the application of recombinant human TRX in COPD management to control enhanced inflammation, whereas other dosage forms, such as inhalation and oral administration of a TRX inducer, must be evaluated in the stable phase of COPD in future studies.

In addition to its therapeutic potential, TRX is also a useful biomarker for several diseases, including COPD. In the human body, the TRX concentration is much lower in the blood (10–100 ng/mL) than intracellularly (1000–10,000 ng/mL) [11]. However, blood TRX levels are elevated in response to various stimuli, including smoking [131]. TRX expression in the sputum is also positively correlated with the degree of hypoxia in patients with COPD [132]. These results suggest that the body attempts to neutralize excessive oxidative stress in COPD lungs by producing TRX. On the other hand, serum TRX levels are significantly decreased during acute exacerbations in patients with COPD accompanied by an increase in 4-hydroxynonenal (4-HNE) in serum [104], which suggests that systemic detrimental oxidative stress occurs by overwhelming the protective effects of TRX during acute exacerbations. Thus, these results suggest that tissue TRX levels are a good noninvasive marker of oxidative stress in patients with COPD and can be used to stratify patients with COPD for the identification of patient subpopulations who will benefit from antioxidant-targeting treatments. For example, a subpopulation of patients with COPD who are more susceptible to exacerbations can be a good candidate for antioxidant treatment [133]. For this aim, a clinical study to determine the baseline of oxidative level in frequent exacerbators is mandatory.

### 4.2. N-Acetylcysteine (NAC)

Despite the protective potential of TRX on COPD pathogenesis, the efficacy of TRX for preventing COPD progression has not been tested in large clinical trials. In contrast, the effects of N-acetylcysteine (NAC) on COPD pathogenesis have been well evaluated in clinical trials. NAC is a thiol compound that shows potent antioxidant effects through the direct and indirect scavenging of free radicals [134]. In CS-exposed rat models, NAC has been shown to inhibit inflammation in small airways and emphysematous changes in the lungs [135,136,137], reversing CS-induced mucous cell hyperplasia to some extent [138]. In addition, NAC attenuates CS-induced antioxidant impairment independently of Nrf2 in mouse alveolar type 2 epithelial cells, a stem cell population in alveoli responsible for emphysema in COPD [139]. Chronic NAC treatment also decreased p16- or p21-positive lung senescence and protected aged mice from the development of emphysema [140]. Thus, these results suggested the protective effects of NAC in COPD occur by reducing oxidative stress damage in addition to cellular senescence.

The clinical efficacy of low-dose NAC was tested in the BRONCUS trial, a randomized placebo-controlled trial, in which 523 patients with COPD received either 600 mg NAC daily or a placebo for three years [141]. This study failed to demonstrate the beneficial effects of NAC treatment in reducing disease progression or the frequency of acute exacerbations, while the subpopulation analysis suggested that NAC treatment might reduce the exacerbation rate in patients without inhaled corticosteroid treatment. In this clinical study, the authors concluded that low-dose NAC (600 mg daily) is not effective against COPD pathogenesis. The efficacy of a higher dose of NAC (600 mg twice daily) was evaluated in a larger randomized placebo-controlled trial, in which over 1006 Chinese patients with COPD were involved receiving either 600 mg NAC daily or a placebo for one year [142]. This clinical study, the PANTHEON study, showed that there was a significant reduction in acute exacerbations of 22% in the NAC treatment groups compared to that in the placebo treatment groups. Moreover, post hoc analysis of the PANTHEON study demonstrated that the reduction in acute exacerbations was the greatest in the subpopulation who were current smokers and did not receive inhaled corticosteroids [143]. A meta-analysis of 13 studies including 4155 patients with COPD, which assessed the efficacy of NAC treatment in preventing exacerbations in chronic bronchitis and COPD, demonstrated that patients treated with NAC had significantly and consistently fewer exacerbations of chronic bronchitis or COPD, especially in patients without evidence of airway obstruction [144]. Consistent with two previous clinical trials, this meta-analysis suggests that a patient suffering from chronic bronchitis with airway obstruction should be treated with a higher dose of NAC (600 mg twice daily) to prevent exacerbations.

Although antioxidants have historically been viewed as cytoprotective against malignancy, recent studies suggest that antioxidants may increase the risk of developing several forms of cancer, including lung cancers, in humans [145,146]. Antioxidants such as vitamin E and NAC markedly accelerated tumor progression in mouse models of Braf^V600E^− and Kras^G12D^− induced lung cancer [146]. Breau M et al. also demonstrated that NAC treatment induces the development of lung adenocarcinoma in 50% of *JunD*-deficient aged mice and 10% of aged control mice while protecting against lung emphysema [140]. These results underscore the importance of contemplating the indication of antioxidant treatment, especially long-term and high-dose treatment in the aged population. Thus, identifying noninvasive reliable markers is critical for the stratification of patients with COPD based on the expected benefits, and the risks of antioxidant treatment must be investigated to maximize their efficacy and to minimize side effects.

### 4.3. The Therapeutic Potential of Current COPD Drugs for Oxidative Stress

There has been little evidence that current COPD treatment such as corticosteroids and long-acting bronchodilators has the beneficial effects on oxidative stress. Systemic administration of corticosteroids is used only in acute exacerbation settings to reduce the treatment failure and improve lung function and breathlessness during exacerbations [147]. Their efficacy mainly depends on their ability to ameliorate enhanced inflammation through HDAC2-mediated anti-inflammatory mechanisms [148]. On the other hand, the chronic daily treatment of systemic steroids is not recommended because of the limited long-term beneficial effects in stable COPD [149] against the long-term adverse effects such as steroid myopathy and osteoporosis [4]. One of the reasons for this is a lack of the ability to control oxidative stress. Although one study demonstrated that inhaled corticosteroid treatment significantly reduced reactive nitrogen species [150], there is no clinical evidence that systemic steroids can ameliorate ROS. Moreover, oxidative stress compromises the anti-inflammatory effects of corticosteroids.

Similarly, long-acting bronchodilators have not been demonstrated to have antioxidant effects clinically, although in vitro experiments using human bronchial epithelial cells and β_2_-longacting/anticholinergic drugs suggested the possibility that the long-acting bronchodilators improve the oxidative stress by inhibiting STAT-1 pathway [151]. Beneficial effects of other adjuvant therapy in COPD such as Roflumilast, a phosphodiesterase-4 inhibitor, and macrolides, antibiotics, have not been shown in the context of oxidative stress despite their clinical efficacy and anti-inflammation effects. These results underscore the necessity of drugs to control undertreated oxidative stress in patients of COPD.

## 5. Conclusions

Studies over the past several decades have demonstrated that endogenous and exogenous ROS play pivotal roles in COPD progression by exaggerating the main pathogenesis, such as chronic inflammation, protease-antiprotease imbalance and detrimental oxidative stress, suggesting that targeting excessive oxidative stress has therapeutic potential in COPD. In addition, noxious oxidative stress contributes to extrapulmonary comorbidities as well, which worsens the quality of life and prognosis of patients with COPD. Unfortunately, current COPD treatment including long-acting bronchodilator and corticosteroids has a very limited ability to improve it. In contrast, antioxidants, such as TRX and NAC, have been shown to ameliorate emphysematous changes in COPD animal models by reducing CS-induced inflammation and oxidative stress. However, it has also been demonstrated in both animal experiments and clinical studies that antioxidant treatment increases the probability of tumorigenesis. This unexpected side effect suggests the necessity to cautiously select the patient subgroup that is the most likely to benefit from antioxidant treatment and has fewer risk factors for lung cancer. To establish precision medicine for patients with COPD in whom oxidative stress is the main pathogenesis, reliable markers in noninvasive specimens, such as TRX levels in the serum, are critical and must be tested in large cohort studies. These markers can also be used to determine the stage at which the intervention is likely to be the most effective to stop or delay the progression of lung destruction due to CS-induced oxidative stress. Although earlier treatment has the greatest therapeutic potential, we also balanced the benefits and risks of antioxidant therapy in the long run. In the context of acute exacerbation settings in which the exaggerated inflammation plays critical roles, TRX is a promising drug candidate because of its anti-inflammatory effects. As we previously demonstrated in the mice model experiment that TRX can ameliorate the enhanced inflammation which systemic corticosteroid fails to regulate [100], the efficacy of TRX during acute exacerbations must be tested in a future study. We believe that the study will open new avenues for COPD treatment. In addition, it will shed light on the possibility that TRX can be beneficial to COVID-19 treatment which shares the pathology such as cytokine storm with COPD exacerbation to some extent.

## Data Availability

Not applicable.
